# Characterizing the cultural landscape of traditional Chinese settlements through genome maps

**DOI:** 10.1016/j.heliyon.2024.e39418

**Published:** 2024-10-16

**Authors:** Hu Zui, Tan Min

**Affiliations:** a National-local Joint Engineering Institute of Digital Conservation& Creative Use Technologies for Traditional Villages and Towns, China; b College of City & Tourism, Hengyang Normal University, Hunan Province of, China

**Keywords:** China, Cultural landscape genes of traditional settlements (CLGTS), Genome maps, Landscape genomes of traditional settlements (LGTS), Landscape genome maps of traditional settlements (LGMTS), Hunan province, Traditional settlements

## Abstract

Plentiful traditional settlements are the key part of Chinese cultural heritage due to their prominent traditional cultural features and rich historical information. The rapidly growing social economy of China raises a pressing need for understanding their rich traditional knowledge and wisdom. In order to explore this issue, Cultural Landscape Genes of Traditional Settlements (CLGTS) has been proposed by Chinese scholars since 2003. Since that, it has been attracting wide attention and has made significant strides in many areas, such as urban planning and the development of tourism. However, CLGTS still faces significant difficulties in addressing the connections between the cultural elements and the entire spatial image of traditional settlements. To fill this gap, this work proposed Landscape Genome Maps of Traditional Settlements (LGMTS) based on CLGTS. First, this work clarified the definition of Landscape Genomes of Traditional Settlements (LGTS); then, this work deeply clarified the connotations of LGMTS, and addressed the relations among CLGTS, LGTS, and LGMTS. Based on these findings, this work further established the classifications of LGMTS and explored its functions. Finally, through a case study, the potential of LGMTS to catch the critical cultural features of traditional settlements was shown.

## 1 Introduction

In China, hundreds of thousands of traditional settlements are often regarded as a critical part of the cultural heritage [1], including “historical and cultural famous villages (or towns) and cities”, “traditional Chinese villages”, “ancient villages”, and “Chinese minority remarkable village (or campus)”. They have been attracting nationwide concern due to their outstanding values and roles in promoting social and economic development [2,3]. For example, some of them with outstanding aesthetic, cultural, economic, ecological, and scientific values [4,5] had been inscribed on the UNESCO (United Nations Educational, Scientific and Cultural Organization) World Heritage List [6], such as Xidi Village and Hongcun Village [7], Kaiping DiaoLou [8], and Nanjing Tulou [2].

China has made significant strides in the preservation of traditional settlements since the 1980s [9]. For example, since its launch, six batches of 8171 traditional settlements have been inscribed on the Chinese Traditional Villages List by the end of 2022. At the same time, significant advances have been also made in theoretical research, such as the evolutionary game model for the protection of traditional villages [10], extension theory for the reconstruction of traditional villages [11], and space gene [[12], [13], [14]]. For example, a number of interesting issues and relevant meaningful findings have been reported over the past decades, including spatial forms and patterns [15], methodologies of classifications [16], evolutionary pipelines [17], spatial image features [18], and spatial layouts [19]. It is clear that the current fruitful findings are beneficial for people to understand the values and active roles of traditional settlements. However, there is still an obvious gap between the existing methods or theoretical models and their entire cultural features. Especially, for a certain traditional settlement, there are still some significant difficulties in using GIS to directly mine its cultural image and reveal the related knowledge [20,21].

Due to the rapidly growing digital economy [22], there is a pressing need for many industries and sectors to seek support and competitive advantages from the cultural resources of traditional settlements. This objectively encourages the Chinese government to further strengthen and promote the preservation of traditional settlements to well inherit and use their ample traditional cultural resources. In effect, the preservation and rational use of traditional settlements becomes a common goal across the world [[23], [24], [25]]. Therefore, multiple disciplines focused on traditional settlements, such as exploring the historical or cultural features [5,26]; a large body of archives reported on how to integrate the research frameworks, knowledge, and methods from different disciplines, such geography, cartography, geographical information science, landscape architecture, sociology and anthropology [27,28]. However, there is still a lack of comprehensive models for addressing the relations between the cultural factors and spatial image of traditional Chinese settlements [18]. This makes it impossible to fully capture the core cultural features of these heritages.

At the current stage, most traditional Chinese settlements have been facing serious difficulties in development, including hollowing [29], decline, damages or continuous encroachment [25] caused by urban expansion [30], and over-development of tourism [30,31]. Note that, there are still some perilous behaviors or activities even though a colossal effort of preservation work on traditional settlements have been performed since the 1980s [9]. One of the main reasons is that the ordinary people have insufficient knowledge of prominent values and roles of traditional settlements in the context of the development of modern society. For example, some villagers living in them maybe dismantle their historic and shabby dwellings with rich aesthetic and cultural values and traditional styles in order to pursuit a modern lifestyle. Hence, this urges us to develop a theoretical model or method to understand the spatial image of cultural landscapes of traditional settlements with the support of GIS [20].

Over the past decade, several research frameworks have been proposed to understand the cultural features of traditional settlements, such as understanding the historic rural settlements at a regional scale [32], clarifying the connections between the historical geographic names and traditional calendars in the whole country [33]. Although they can help understand the development or evolution of historical rural settlements, or partly describe the corresponding spatial-temporal information, these frameworks still lack the capability to capture the critical cultural features of traditional settlements from the perspective of geography.

In order to uncover the complex associations among cultural elements and the correlations between cultural factors and the entire spatial image of traditional settlements, Liu presented the concept of Cultural Landscape Genes of Traditional Settlements (CLGTS) in 2003 [34]. Through connecting multi-disciplinary methods or models, such as biology, architecture, geography [35], CLGTS lays a theoretical foundation for understanding the core features of cultural factors of traditional settlements. Since then, it has been widely used in many application fields, such as tourism development planning of traditional settlements [34], cultural zoning of traditional settlements in China [36], identification of cultural features of Hakka's traditional settlements in China [37], and visualization of the cultural factors of traditional settlements [1]. However, for a certain traditional settlement, CLGTS is still difficult to capture the correlations between this settlement's cultural elements and its whole spatial image.

The genetic analysis methodology was first proposed by Johannsen in 1909 [38]. It has been widely used to cope with the issues of different fields, such as social economic-genetic theory for corporate corruption [39], human cognitive behaviors [40], social-cultural co-evolution relationships [41], quantitative analysis of cultural evolution trend [42], and algorithms improvement [43]. The genetic analysis methodology and biological gene theory has had a profound impact on natural science, computer science [44], and sociology; genetic analysis methodology therefore vastly extends the scientific boundaries of humanity [42].

Although it is very mysterious, the nature of life can still be understood well with the help of contemporary bioinformatics. For example, genome maps can map the core features of genes through spatial sequences, position, physics, chain, as well as heredity information. According to bioinformatics, genome maps can well depict the pivotal genetic features, including precise positions, ingenious spatial structures, and accurate identify self-duplication, because the key genetic characterizations are well organized and visualized through rational use of the principles and methods of geometry, mathematics, and computer science. Similar to the biological genetic features, the features of social cultures can also be depicted or clarified by using the principles and methods of genome maps, such as culturomics [42]. This significantly implies that the complex correlations between the cultural factors of traditional settlements and their spatial images can be well clarified through referencing genome maps. In order to explore this issue, through bridging the methods and principles of genome maps, we propose Landscape Genome Maps of Traditional Settlements (LGMTS) based on CLGTS. Hence, this work mainly focuses on exploring the conceptual framework of LGMTS and the related theoretical significance.

The remainder of this work is structured as follows. Section 2 overviews CLGTS, including definition, main features, identification rules and methods. Section 3 details the definition and principles of LGMTS. Section 4 elucidates the main processes and methods for an instance of LGMTS through a case study. Section 5 discusses the theoretical implications of LGMTS. Section 6 contains the main conclusions of this work. The final section outlines a few key issues in the future to further promote LGMTS.

## 2 An overview of the theory of CLGTS

### 2.1 Definition

The organic gene is a functional unit with rich genetic information. The natural features of an organic gene can be described or portrayed through its unique structures, precise self-replication, stable expressions, as well as functions. In fact, the constituent elements or influence factors of social cultures are similar with organic genes, such as maintaining their unique connotations or forms. So do the traditional settlements [35]. For example, the spatial layout of Zhangguying Village, located in Yueyang county of Hunan Province, China, has been maintained until now, and there have been no apparent changes since its foundation in ancient [1]. Note that the scholars have observed the similar attributes and features between the cultural factors of traditional settlements and organic genes. Liu first proposed the concept of CLGTS to describe the key features of cultural factors of traditional settlements and revealed the pertinent correlations in 2003 [34]. According to Liu, only when the following requirements are met can a cultural factor of a certain traditional settlement be defined as CLGTS: (i) it can be inherited from generation to generation in traditional settlements; (ii) it can help people distinguish its affiliated settlement from the others; (iii) it is the smallest and indivisible unit with rich social, cultural, and historical information; and (iv) it can be recognized without uncertainty, imprecision, and ambiguity.

### 2.2 Main features

CLGTS is conducive to the understanding of important cultural features of traditional settlements since it provides a potential quantitative method for examining these features and attributes or properties from a perspective of geographic information [20].

Above all, its concept is based on a comprehensive understanding and a deep insight into Chinese history and traditional cultures [35]. From the perspective of cultural geography, it can be thought as the basic unit of traditional cultural and historical information, such as traditional knowledge, social ethic, values, religions, and politics. It is important to note that all the information carried by CLGTS is an indispensable part of traditional settlements. Hence, it can effectively map and describe the relations between the natural environment and social cultures on a micro-scale.

Secondary, it deeply refines and abstracts the symbolic properties and connotations of cultural landscapes of traditional settlements. For example, for a certain traditional settlement, its spatial image [18] can be drawn and outlined through its spatial layouts and related auspicious geomantic (or fengshui) meanings, and the spatial structures of alleys and streets. It can help capture the salient cultural features of a given traditional settlement.

Additionally, it effectively generalizes the attributes of carriers and cultural significance. In effect, we can use geometry, mathematics, and GIS to thoroughly examine CLGTS according to natural environments, resources, constituent elements or influence factors, and buildings, and construction technologies. It provides a dual description of the dominant cultural characterizations of traditional settlements by ‘shapes-mathematics-rules’ [43].

Finally, CLGTS can be divided into two categories because the cultural factors of traditional settlements can be divided into tangible and intangible categories. This means that we can model some quantitative attributes or shed light on some outstanding qualitative properties of CLGTS. The tangible elements or factors of traditional settlements usually have physical carriers and are easy to be quantitatively described or depicted. On the other hand, the intangible elements or factors of traditional settlements can be usually drawn through cultural factors, elements, phenomena, and events without physical carriers. As a consequence, CLGTS is helpful in building a comprehensive framework by combing the quantitative and qualitative methods.

From the above, CLGTS can be treated as a paramount parameter in dissecting the core cultural features of traditional settlements.

### 2.3 Identification rules

The identification rules for CLGTS were developed after it was first proposed in 2003 [35]. They are namely inherent uniqueness, external uniqueness, local uniqueness, and superiority. They are widely used, such as the identification of dwelling features of Hakka's traditional settlements [37], and the recognition of spatial patterns of traditional settlements in Hunan Province [15].

First of all, inherent uniqueness highlights that a CLGTS is a unique cultural element or factor, and it is only investigated in its affiliated traditional settlement. According to this rule, for a given CLGTS, if it is recognized in a traditional settlement, it should not be concerned in the other traditional settlements. Because a CLGTS is the most outstanding cultural feature or attribute for a given traditional settlement and it can help discriminate its affiliated settlement from the others.

Secondly, external uniqueness mainly highlights the unique features, attributes, appearances, or properties of CLGTS in the external conditions of existence and environments. Through this rule, a CLGTS can be recognized in terms of its owned features or properties. Obviously, for a certain CLGTS, this rule suggests that its external features or conditions of existence can only be found in its affiliated settlement rather than the others.

Thirdly, local uniqueness emphasizes the most critical features of a CLGTS. According to this rule, the nuances or local features of a cultural factor can support the identification of CLGTS. Obviously, a CLGTS can be accurately determined through its local salient features or properties, even if it has highly similar features to other cultural factors. For example, for a given region, although some cultural factors with highly similar and even same features may simultaneously exist in a couple of traditional settlements, the pertinent CLGTS of a traditional settlement can be distinguished according to their critical local features or details.

Lastly, superiority stresses the use of holistic viewpoint to determine CLGTS in situations where the above rules still remain difficult. In a given area, if a cultural factor is present in several traditional settlements, how can we determine which traditional settlement can claim it as a CLGTS? According to the superiority rule, a cultural factor of a traditional settlement in this area can be identified as a CLGTS only if that cultural factor is more prominent than that of other settlements. In addition, there are a few other crucial attributes which can help determine CLGTS, such as importance, integrity, and predominance.

Note that the above rules are not separate or opposite; on the contrary, they are supplementary to each other (Table 1).

**Table 1:** The four identification rules for CLGTS.

Rules	Interpretations	Notes
Inherent uniqueness	A CLGTS of a given traditional settlement is the unique cultural factor that can discriminate its affiliated settlement from others.	According to this rule, people can recognize a traditional settlement through its CLGTS.
External uniqueness	It emphasizes the owned attributes and features of a CLGTS.	In terms of this rule, a CLGTS of a given traditional settlement can be recognized through its external appearances or conditions.
Local uniqueness	A CLGTS can distinguish itself from the others through its local unique attributes or nuances.	According to this rule, we can recognize a CLGTS through its local attributes or nuances if there are many cultural factors with similar features.
Superiority	A CLGTS can distinguish itself from many cultural factors with highly similar characteristics through the entire features.	For a given traditional settlement, its CLGTS is the most outstanding cultural feature than the others.

For a given traditional settlement, to fully understand its CLGTS, it is necessary not only to translate its appearance features, but also to examine its inherent characteristics such as historic, natural, cultural, ethic and religion. The above identification rules are rather helpful in capturing the nature of CLGTS [20]. Inherent uniqueness mainly highlights the inherent natural features of CLGTS and we can examine a CLGTS through its origination. External uniqueness can provide rich information of a CLGTS through its appearance features. At a regional scale, the uniqueness and superiority provides a potential approach to solve the dilemma of multiple highly similar cultural factors existing in some traditional settlements at the same time. Through the above, there is a parallel connection between inherent uniqueness and external uniqueness; and there is an incorporation connection between local uniqueness, superiority and inherent uniqueness, and external uniqueness [36]. On the whole, superiority can incorporate inherent uniqueness, external uniqueness, and local uniqueness. These connections among the identification rules can help people determine CLGTS for a given traditional settlement, because they have laid a solid foundation for the identification methods.

### 2.4 Identification methods

Traditional Chinese settlements usually have a long history and have accumulated rich traditional knowledge, cultures, and skills, such as traditional regimes, politics, ethic, religions, customs, human-land relation, as well as arts. It is worth stressing that the cultural factors, elements or phenomena of traditional settlements can be classified into tangible and intangible cultures. Therefore, for a given traditional settlement, it is difficult to determine its CLGTS from complex cultural carriers, and requiring people to deeply dissect its core characterizations, and even repeatedly compare different carriers or elements. At the same time, the whole analysis process should conform to the integrity principle of CLGTS [37], which highlights the close co-relations between CLGTS and other cultural factors or phenomena. In order to unambiguously recognize CLGTS, Shen et al. developed four methods in 2006, namely, element analysis, feature pattern, structure analysis, and text description [36].

Element analysis means that we can determine whether a traditional cultural factor is CLGTS in terms of some crucial attributes of its components, such as appearances, shapes, colors, textures, or symbolic connotations, cultural implications, functions. To a certain degree, for a given traditional settlement, this method suggests that we can determine its CLGTS through recognizing the related crucial cultural characterizations based on the principle of cultural diversity [3].

Feature pattern means that we can recognize CLGTS according to the structured delicate patterns with rich cultural significance. In practice, we can extensively collect and document the feature patterns of cultural factors and elements of many typical traditional settlements. This can help determine CLGTS for a given traditional settlement. For example, many Chinese minorities have their own totems with explicit patterns, and this cultural phenomenon can be usually observed through the traditional buildings of campus, decoration styles of traditional buildings, religions, and customs.

Structure analysis states that we can distinguish CLGTS according to the cultural factors with stable and meaningful spatial structures or layouts. For example, the site and spatial layouts of many traditional settlements usually conform to the ancient geomantic principles; so, each traditional settlement has its own special structures and spatial layouts with propitious willingness. We therefore can determine CLGTS through understanding the core features and properties of spatial structures.

Text description implies that we can use text to define and describe CLGTS through the natural features and attributes of cultural factors if it is difficult to determine CLGTS through the above identification methods. In traditional settlements, there are usually many paramount intangible cultural factors and it is difficult to directly describe them through structures or elements, and patterns.

Note that, the above identification methods have been proven by a couple of research works, such as zoning the traditional settlements in China [45], and determining the spatial patterns of CLGTS in Hunan Province, China [15].

## 3 Landscape Genome maps of traditional settlements (LGMTS)

### 3.1 Genome maps

According to bioinformatics, an organic genome is usually defined as the collection of all the genetic information of its DNA, such as the spatial structures of arrangements, heredity information coding, expressions, functional features, and heredity attributes. This genetic information can be recorded and expressed through graphs, figures, diagrams or curves with the help of mathematics, geometry, and computers. Obviously, the genetic information of an organic genome and its related expressions constitute the genome maps. Currently, genome maps mainly focus on how to mark spatial positions or make clear the patterns of distribution and arrange sequences of genes through chart, graph, figure and mathematics curves [46]. The genome maps therefore are widely used to analyze the sequence properties, position the spatial coordinates, and elucidate the particular functions of genetic units [47].

From the biologic perspective, the following important features of genome maps are clear: (i) they have their own physical carriers; (ii) they can feature their specialized functions and mechanisms; (iii) with the help of math, geometry as well as informatics, they can visualize the key information of genetic units such as spatial curve [48], 3D structures and textures [49], and informational reflect [50].

However, in many social, economic and humanity domains, genome maps can also play a full role in modelling or analysis and are seen as a useful scientific method, because they connect several disciplines such as math, physics, chemistry, geometry, and informatics. For example, genome maps are introduced as an approach to modelling the evolution pattern of human cultures [42]. In addition, from the methodological perspective, some merits can be easily drawn. From the viewpoint of hierarchy and integrity, they can deal with the functions, spatial structures, and phenotype of genetic information within the comprehensive analysis framework, while not neglecting the co-relations between different genes [42]. Moreover, they can also represent the key features and rules of genetic information through understandable curves, charts, graphs, figures or diagrams. Genome maps hence can distinguish or label a gene through combining the information technical methods. With the support of informatics, genome maps are usually seen as a popular tool that can play a meaningful role in understanding the core cultural features of traditional settlements.

### 3.2 Landscape genomes of traditional settlements (LGTS)

Similar to the biological genome, for a given traditional settlement, all the constituent elements of its cultural landscape can make up a spatial image [37,51]. For example, for a certain traditional Chinese settlement, all the constituent elements of its cultural landscape are rich in geomantic meanings or wills, such as Shuikou, public buildings, streets and alleys, and plazas. What deserves to be mentioned is that it is difficult to fully understand the geomantic meanings of all the constituent elements of cultural landscapes of traditional settlements if we examine each element separately or neglect the co-relations between different elements, because a single cultural element cannot exist separately, but rather is connected with the others. This suggests that the natural features of cultural landscapes of traditional settlements can be deeply understood through treating all the constituent elements as a whole [37]. For example, the traditional settlements in the region of Yangtze River Delta are famous for their owned arch bridges, water streets, and traditional ancient yard; they are very popular with the tourists across the world because their scenes are very similar to the ancient Chinese poem “little bridge-flowing water-dwellings” [18]; hence, only by examining the cultural implied meanings between bridges, rivers and dwellings through the integrity principle of CLGTS [37] can we really grasp such poetic image.

**Fig. 1:**
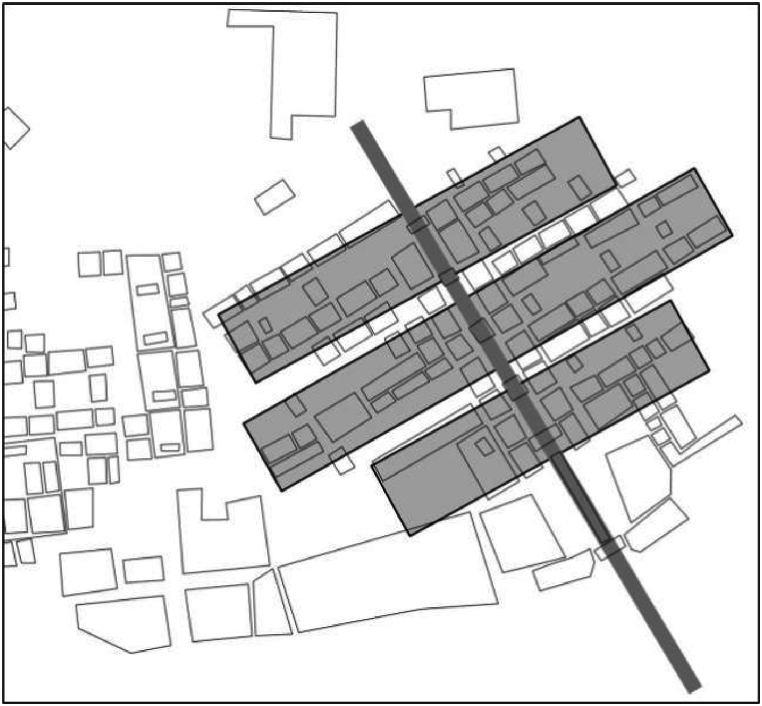
The layout of Dang Gate of Zhang Guying Village. Zhang Guying Village is located in the Yueyang County of Hunan Province, China. The layout of Dang Gate is designed as a Chinese character “丰”. It means a good harvest for villagers and longevity for the old.

Through the above, this work proposes Landscape Genome of Traditional Settlements (LGTS) to understand the natural features of traditional settlements based on CLGTS. According to the integrity principle of CLGTS [37], LGTS mainly focuses on the following two aspects: (i) how to outline the entire features which contain the critical characteristics and historical information at a whole level; and (ii) how to extract the core properties from the total CLGTS based on the former step. For a given traditional settlement, more correlations or connections or meaningful rules can be observed by analyzing its CLGTS at a whole level than by analyzing each CLGTS one by one. In other words, this means that we can deeply draw the correlations, rules and core features of cultural landscapes of traditional settlements according to the related LGTS. The entire spatial layouts of many traditional Chinese settlements are designed on the basis of ancient geomancy and are rich in traditional cultural meanings. This suggests that LGTS is helpful to understand the spatial image of cultural landscapes of traditional settlements. Here, we take the spatial layouts of Dang Gate in Zhangguying Ancient Village as an example (Fig. 1). Zhangguying Ancient Village is located in Yueyang County, Hunan Province, China. Dang Gate is the most important building group of traditional courtyards in the village and is famous for its owned layout “five dooryards in one line”. It has a distinct symmetric axis, designed along the five dooryards (Fig. 1). Each house shares the same construction style and is arranged on both sides of the symmetric axis. Note that Dang Gate's houses congregate into three rows along the symmetric axis. The whole spatial layout therefore likes a Chinese character ‘丰’. This spatial layout is rich in traditional cultural meanings, such as a good harvest every year, prosperous development for the whole village and longevity for the old.

### 3.3 Landscape Genome maps of traditional settlements (LGMTS)

Generally speaking, maps are usually viewed as a valid scientific method and even as a methodology in many scientific fields [42]. Maps are usually employed to uncover and visualize the natural laws of complex temporal spatial questions. In fact, many methods from different disciplines are integrated into the maps in order to analyze, simulate, and predict the rules and laws of the research objects or questions through charts, graphs, tables, curves, diagrams or figures. So, from the humanities to the arts, and from natural science to engineering science, maps are widely used in many areas to resolve different issues. Obviously, maps are a valid and scientific approach to portray and express the natural features of LGTS. Through connecting the principles of genome maps of bioinformatic and culturomics [42], this work presents the concept of Landscape Genome Maps of Traditional Settlements (LGMTS) in order to deeply capture the core features of cultural landscapes of traditional settlements.

The concept of spatial image of traditional settlements was first presented by Liu and Dong [19] by referencing the concept of urban image. It provides an entire perspective for analyzing the historical and cultural information of CLGTS and drawing the natural features of traditional settlements. In essence, for a given traditional settlement, its CLGTS can be recognized through the identification methods, including element analysis, feature pattern, structure analysis, and text description; and then, people can analyze and draw the related connections and correlations, as well as patterns. Hence, it is urgent to find an appropriate method to express the critical features of CLGTS on the basis of LGTS. Note that maps can well portray the rules, spatial patterns, spatial associations and connections in geographic fields [51] because they can integrate the expression methods (shapes), reasons (mathematics), and rules (interpretations). Besides, maps can extend the scientific boundaries of modelling complex cultural phenomena [42] for featuring LGTS.

**Fig. 2:**
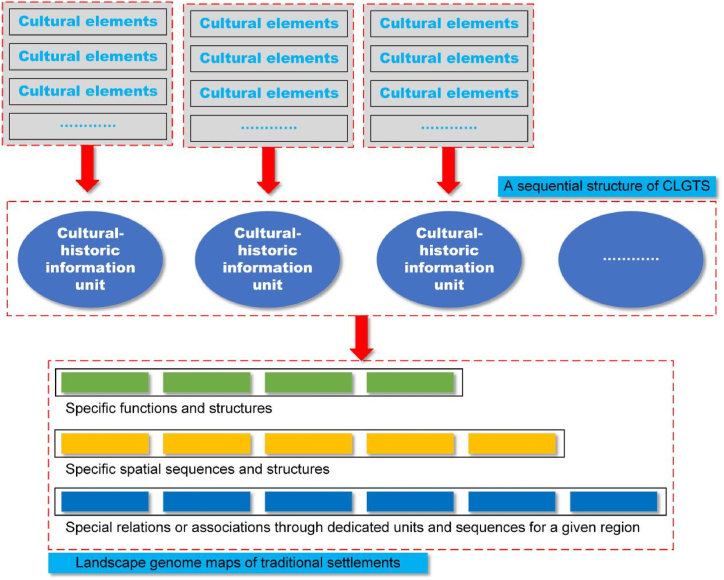
The conceptual diagram of LGMTS.

In terms of the above, LGMTS is a scientific method that uses the principles and methods of bioinformatics to analyze and translate the natural features, rules and connotations of spatial layouts, structures, forms, and design of LGTS with maps (Fig. 2).

### 3.4 Correlations among CLGTS, LGTS and LGMTS

**Fig. 3:**
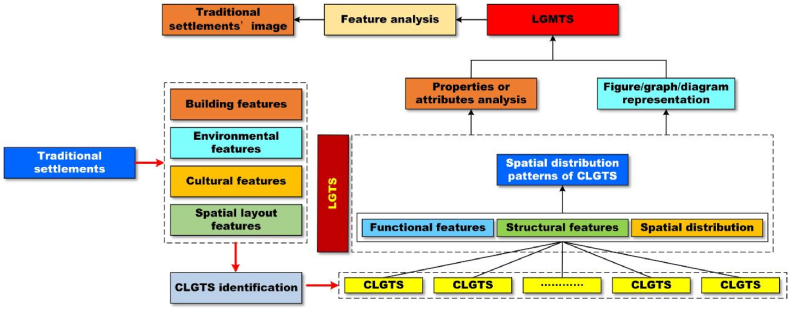
The relations among CLGTS, LGTS, and LGMTS.

In order to address the critical features of cultural landscapes of traditional settlements, it is necessary to determine the correlations among CLGS, LGTS and LGMTS. Here, the following correlations are outlined (Fig. 3).

In the first place, for a given traditional settlement, its LGTS usually includes all of its CLGTS. In other words, a CLGTS has its own unique spatial positions and undertakes certain specific functions. It is noteworthy to stress that the spatial distribution of a CLGTS is constrained by some important elements of traditional settlements, such as the surrounding terrain of settlements, rivers, public buildings, and ancient planning ideas. In fact, for a given traditional settlement, the distribution positions and ways of all of its CLGTS form a unique spatial layout or pattern, which has rich traditional cultural connotations. Therefore, from this point of view, for a given traditional settlement, all of its CLGTS and the corresponding spatial distribution layout or pattern together constitute its LGTS. There is an obvious inclusion relationship between CLGTS and LGTS.

Besides, LGMTS clarifies the most noteworthy cultural features and properties of historical and cultural information of LGTS by quantitative and visual methods. For example, for a given traditional settlement, in order to deliver the core characterizations of its LGTS, people can organize its LGMTS through charts, tables, graphs, and curves. On the other hand, for some given traditional settlements, LGMTS can also be used to observe the critical features of their spatial image. It is clear that LGMTS is the most appropriate scientific method to reveal the important features of historical and cultural information of LGTS.

Then, for a given traditional settlement, LGMTS mainly focuses on the overall features of its all CLGTS. LGMTS can depict the key features of CLGTS by the visual scientific diagrams or tables and figures. Therefore, LGMTS is helpful to understand the entire features of spatial image of a certain traditional settlement.

### 3.5 A brief classification for LGMTS

Establishing an appropriate classification can help us gain insight into the nature of LGMTS. By referencing the classification method of Geo-Tupu [52] and bio-genome maps [46], this work develops a concise classification for LGMTS (see Table 2). LGMTS is divided into three parts: spatial sequence atlas, arrangement schematic atlas, and spatial pattern atlas, respectively. As a matter of fact, this classification is also a possible approach to establish LGMTS for a given region.

**Table 2:** A brief classification for LGMTS.

type	objectives	expressions
spatial sequence atlas	a given traditional settlement	the distribution rules, regulars, and features and the related entire functions of all CLGTS.
arrangement atlas	a given region	the common properties and features of the same type of LGTS.
spatial pattern atlas	a given region	refining and expressing the spatial distribution patterns or laws of every type of LGTS.

For a given traditional settlement, the spatial sequence atlas mainly describes the corresponding spatial distributing rules and features by defining it's CLGTS as a whole. However, it is important to stress that the arrangement schematic atlas and spatial pattern atlas are mainly used to deliver the core features of all LGTS in a given region. For a given region, only after we have built the spatial sequence atlas of each traditional settlement can we begin to construct the arrangement atlas. Then, each LGTS in this region should be examined in order to draw the common rules and properties, and people can make the diagrams, figures, forms or graphs of CLGTS schematics of each traditional settlement in this region. Lastly, people can establish the arrangement atlas through the common features and rules of these diagrams, figures, forms or graphs. It is clear that the arrangement atlas can translate the most important and noteworthy features of LGTS at a regional scale. For a given region, people can determine the types and features of all LGTS according to the related arrangement atlas. Based on the above results, people can make the final spatial pattern atlas.

### 3.6 The functions of LGMTS

People can explore and draw the critical rules and laws of traditional cultures on a regional scale by constructing the related LGMTS. Here, this paper explores several promising functions or features of LGMTS. (i) It can help people catch and determine the CLGTS distribution patterns of a given traditional settlement. For example, if the CLGTS distribution pattern of a given traditional settlement is unclear, we can figure out this issue by comparing its sequence atlas with those of other settlements. (ii) For a given region, it can help people grasp the common properties and attributes of spatial layouts of LGTS. For example, in a given region, the rules and laws of spatial structures and layouts of all traditional settlements in this area can be observed and summarized according to the spatial sequence atlas and pattern atlas of LGMTS. (iii) For a given region, we can recognize the cultural features of a traditional settlement through comparing the LGMTS of each traditional settlement. For example, through a thorough analysis of related spatial sequence atlas and arrangement atlas, we can compare the cultural characterizations of a traditional settlement with those of another one. (iv) It provides a useful toolkit to explore the differences between various LGTS in different regions. For example, the nuances of traditional quadrangle courtyards in different regions can be figured in terms of its spatial sequence atlas of CLGTS.

Note that the above does not incorporate all. More functions will be investigated and reported with more research and applications in future.

## 4 A case of LGMTS in Hunan Province

### 4.1 Research areas and materials

Hunan Province is located in south-central China, in the middle reaches of Yangtze River and south of the Dongting Lake (the second largest freshwater lake in China). Xiangjiang River runs through the whole of Hunan Province from south to north. Hunan Province covers an area of 108°47′ to 114°15′E and 24°38′ to 30^0^08′N, about 210,000 square kilometers. It divides itself from Jiangxi Province by the Mubu Mountain, and the Wugong Mountain nears to east. It also neighbors Guizhou Province through the eastern edge of the YunGui Plateau. Chongqing Municipality is located in the northeast of Hunan Province, bounded by the Wuling Mountain. In the south, Guangdong Province and Guangxi Autonomous Region are bounded by Hunan Province. And in the north, the Dongting Lake separates Hunan Province and Hubei Province on a wide plain. Hunan Province spans more than 667 km from east to west, and covers about 774 km from north to south. Hunan Province has a typical subtropical monsoon climate [16], with sufficient sunshine and rainfall. Hence, it is very popular with tourists because of the four distinctive seasons and hot weather and rain during the same period.

Note that Hunan Province has a very long history. In terms of the modern archeological findings, the trace of the ancient human in Hunan Province can trace back to the Old Stone Age. In the era of the ancient Xia Dynasty, Shang Dynasty, and Xizhou Dynasty, Hunan Province was ruled by Jingzhou State. From Chunqiu to Zhanguo, Hunan Province was governed by Chu Kingdom. In Qing Dynasty, Hunan Province was formally confirmed as an administrative district. Since then, it has been in use to now [53].

**Fig. 4:**
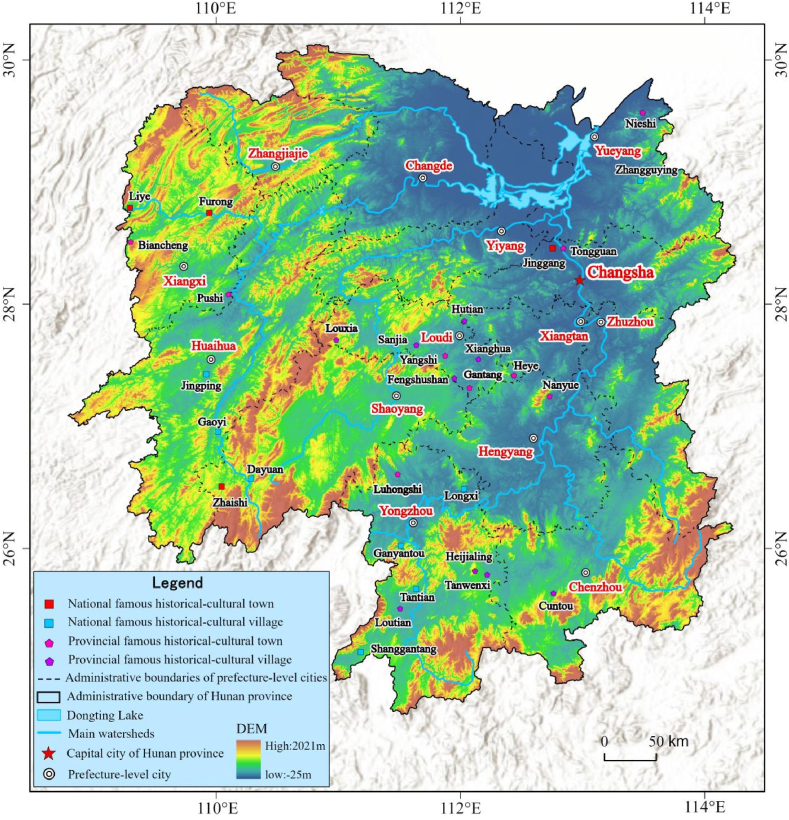
The distribution of sample traditional settlements. To the end of 2022, Hunan Province has 4 national historic-cultural towns, 9 indigenous historic-cultural towns, 2 national historic-cultural villages, and 15 local historical-cultural villages.

To sum up, Hunan Province fostered a tremendous amount of settlements in history because of good environmental conditions and resources, and deep history and traditional cultures, and most of them have been inherited to now. According to the Culture and Tourism Bureau and the Dwellings and Rural and Urban Construction Bureau, Hunan Province remains 30 well-preserved national and provincial historic-cultural towns/villages [15]. This work thus takes them as samples (Fig. 4) to establish LGMTS for Hunan Province.

This work collected the prerequisite materials and data. We first collected books on the history and cultures of Hunan Province, namely, Ancient Towns of Hunan [54], Local History Atlas of Hunan Province [55], and A Historical-cultural Geographic Research on Hunan Province [56]. Then, we built a set of street maps of all samples on a scale of 1:1000. We employed Google Earth Engine to collect the remote sensing images of all samples and used GoodyGIS to capture the vector data of each sample. In addition, we also downloaded the basic geographical information datasets of Hunan Province on a scale of 1:500,000, and Digital Elevation Models (DEM) on a scale of 1:250,000 through the website of the National Geographical Information Center of China. Next, we normalized the GeoReferenced System as CGS-84 of China, and the projection as Gauss-Krigve, and finally organized all the materials into a geodatabase through ArcGIS 10.1 software.

### 4.2 Methods

#### 4.2.1 Identification index system of CLGTS

In fact, an appropriate index system is very conducive to identify CLGTS [15]. Note that, according to the definitions and scientific connotations of CLGTS, the core features and properties of cultural landscapes of traditional settlements can be addressed by four aspects, including cultures, environments, architectures, and spatial forms. Some important traditional cultural factors have been proposed to help recognize CLGTS [42], such as the styles of traditional dwellings, totem, public buildings, environmental factors, and spatial layouts. In addition, some influencing factors that can affect the identification results of CLGTS have been explored [45], including psychology factors, biologic factors, aesthetic factors, environmental factors, cultural factors, and temporal factors. Hu et al. developed an identification index system in 2013, which is composed of 14 indicators in 4 categories, namely, buildings, traditional cultures, environments, and entire layouts (Table 3) [15].

**Table 3:** Identification index system for CLGTS.

Class	Features	Cultural factors
buildings	building features	roof shape
		figure of gable wall
		house facade
		house elevation
		decorations
		materials
	public buildings	main public spaces
traditional cultures	totem	totem marks
	religion	religion/belief
	customs	daily customs
environments (elements)	landform	landform
	river/watershed	river
entire layouts	forms	spatial form
	structures	spatial structures

In order to deeply address this index system, based on Table 3, a couple of critical features are detailed as follows.

To begin with, buildings consist of two parts. The first part includes roof shape, figure of gable wall, house facade, house elevation, decorations, and materials. The last part merely means the public buildings, which is usually treated as the main public places for the important public activities in the traditional settlements. In addition, in some circumstances, the public buildings can also be viewed as the symbol of traditional settlements. For example, in a Dong Ethnic traditional campus, a tall drum-tower is usually situated in the center and thus undertakes many important public activities.

Afterwards, the core features of traditional cultures can be drawn through totem, religions, and traditional customs. These cultural factors can effectively smooth away the difficulties and reduce the complexities for the identification of CLGTS.

Once again, environments can comprehensively highlight the important characteristics of the sites where the traditional settlements are located. This case mainly determines the connections between the environments and traditional settlements through landform and rivers. Because that, in ancient China, people always try to combine the landform features with the rivers in order to create ideal settlements with rich geomantic implies.

Ultimately, entire layouts mean that the spatial layouts of traditional settlements designed by the ancient people according to the principles and methods of geomancy are usually full of auspicious cultural meanings. This can be observed through the sites of traditional settlements and related patterns of their spatial layouts. Note that the entire layouts often include the ancient natural philosophy and survival wisdom [[57], [58], [59]]. This case mainly discriminates the pivotal features of entire layouts of traditional settlements through spatial forms and structures.

In ancient China, with the migrants moving from north to south, the survival and constructions knowledge and skills had been propagated. For example, Hakka culture witnesses the forms of Hakka's traditional architecture, such as Tulou [60]. However, there are significant differences between the traditional settlements of the north and the south, because the ancients adapted their knowledge and skills to new environments as they migrated. These adaptions or changes eventually were recorded or reflected by the cultural features of traditional settlements. Besides, the ancient building materials often originated from the surrounding environments and resources. As a whole, the above are beneficial to develop an identification index system of CLGTS.

#### 4.2.2 The building workflow for LGMTS

This work determined all CLGTS of each sample according to the above index system (Table 3). Based on the identification results, this work further built the sequence atlas of each sample. Then, this work established the arrangement atlas via comparing the sequence atlas of each sample. The arrangement atlas and sequence atlas can display a good play in exploring the spatial pattern atlas.

**Fig. 5:**
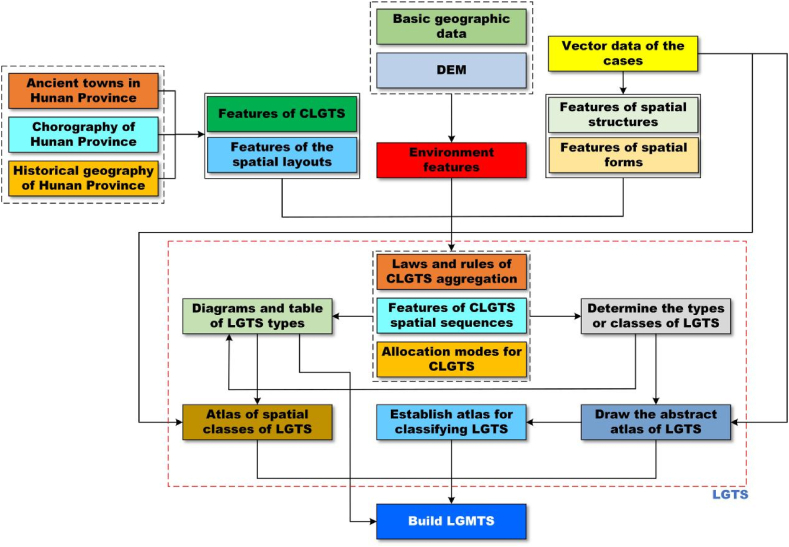
Workflow chart. The workflow effectively guides the whole research work through 5 steps.

Overall, this work developed a scientific workflow (Fig. 5) to carry out the case study of LGMTS in Hunan Province.

**Fig. 6:**
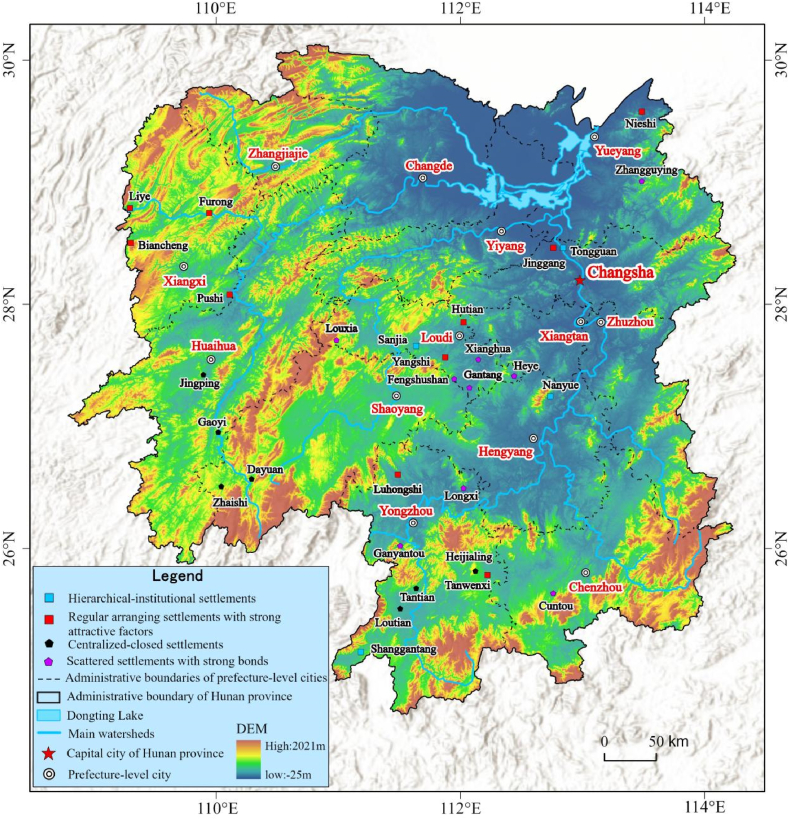
The spatial pattern of LGTS of Hunan Province. There are four types of LGMTS, such as hierarchical-institutional settlements, regular arranging settlements with strong attractive factors, centralized-closed settlements, and scattered settlements with strong bonds, respectively.

This workflow is composed of five steps: (i) according to the materials, we determine CLGTS of each sample and the related LGTS; (ii) we construct the sequence atlas of each sample based on the results of the first step; then, we define the related arrangement atlas (Table 4) through classifying the sequence atlas of each sample; (iii) we feature every type of LGTS through graphs, figures or diagrams and draw the related critical features (also see Table 4); (iv) we further make a table to demonstrate the crucial properties and features of every type of LGTS (Table 4); and (v) after we combine the geographic datasets and different LGTS of all samples and organize these materials into ArcGIS software, we finally establish the spatial pattern atlas (Fig. 6).

**Table 4:** The main features of the arrangement atlas of LGTS of Hunan Province.

class	diagrams for the typical sequences	critical properties or features	abstract graphs
hierarchical-institutional settlements		Nanyue Town is a typical representative of hierarchical-institutional settlements. The Great Temple is the most noteworthy building in Nanyue Town and is famous for its features of clear hierarchy, regular and strict symmetry. The Great Temple is comprised of two parts. The first part is Taoism temple group, which includes 8 Daoguan. The last part is Buddhism temple group, which consists of 8 Buddhism temples. There is a symmetric axle through the entire Great Temple, and the Taoism temple group strictly opposites to the Buddhism temple group along the symmetric axle. The spatial layout of the Great Temple delivers that there is an equal and existence in harmony relationship between Taoism and Buddhism of Nanyue Town but not conflict.	
regular arranging settlements with strong attractive factors		Jinggang Town is a regular arrangement settlement with strong attractive factors, because it is located in the lower reaches of Xiangjiang River, also in the intersection of the Weijaing River and the Xiangjiang River. In ancient times, Jinggang Town was famous for its convenient transport location and shipping. So, it built a good port in ancient times. In history, it had been one of the four major rice trading centers in Hunan Province. The most of its ancient streets are regularly arranged along the shipping channels and neighbored to the port. Importantly, the entire layout of Jinggang Town characterizes as “eight street- four alley- seven harbor”. Tourists often surprise at the diversities of traditional cultures in Jinggang Town.	
centralized-closed settlements		Gaoyi Village is the most typical centralized-closed instance in all samples. Its development core is Wutong Temple that is located in the center of the village. At the same time, Gaoyi Village is composed of five blocks (natural villages), which are all around the Wutong Temple. As a whole, its spatial layout is like five blooming wintersweet flowers. Gaoyi Village is situated in a semi-open basin which like an ancient Chinese Taishi Chair, and the Wushui river blocks the open direction of the basin.	
scattered settlements with strong bonds		Ganyantou Village is a typical example of scattered settlements with strong bonds in this work. It is famous for its villagers from the same clan and it has apparent traditional Chinese cultures of clan-blood settlements. Zhoujia Big Yard is the most important public building of the village. Although the whole village is very scattered, its entire layout is shaped like a Dipper.

### 4.3 Characterizations of LGMTS of Hunan Province

The LGMTS of Hunan Province includes three parts, namely, the sequence atlas of each sample, the arrangement atlas, and spatial pattern atlas. The arrangement atlas delivers the crucial features of LGTS of all samples. The spatial pattern atlas addresses the distribution features and rules of every type of LGTS in Hunan Province.

#### 4.3.1 Features of the arrangement atlas

In terms of the arrangement atlas, we can draw the features of all samples' LGTS and this can help us further establish the classifications of Hunan Province's LGMTS. According to the arrangement atlas, this work distinguishes four types of LGTS, including hierarchical-institutional settlements, regular arranging settlements with strong attractive factors, centralized-closed settlements, and scattered settlements with strong bonds, respectively. In order to make clear the core features of all LGTS, we make diagrams to depict the rules of CLGTS arrangement and the related spatial patterns. Through the arrangement atlas, the following features are observed.

To begin with, the layout of hierarchical-institutional settlements has prominent symmetrical features. In this type of settlement, CLGTS are often arranged along either side of the symmetrical axis. In this case, Nanyue Town, Tongguan Town, Shanggantang Village, and Sanjia Village are typical samples of hierarchical-institutional settlements.

Additionally, the regular arranging settlements with strong attractive factors are usually famous for their convenient transportation. This type of settlement has been the key regional center of commerce and trade in history since the convenient transportation could bring enough goods, customers, and merchants together. It is obvious that convenient transportation is one of the most important driving forces for promoting the development of such settlements. In this type of settlement, many cultural factors or elements, such as restaurant, inn, and traditional Chinese private bank, are attracted and placed along the key communication lines. In this case, the following samples, including Jinggang Town, Yangshi Town, Nieshi Town, Liye Town, Furong Town, Biancheng Town, Gaosha Town, Tanwenxi Village, Pushi Town, and Luhongshi Town, are recognized as the typical representatives of this type.

Next, the type of centralized-enclosed settlements usually has two stark features. First, such a settlement of this type has distinct boundaries. Note that, the boundaries of this type may be composed of the man-made constructions or buildings such as walls, towers, or the natural objects and environmental elements such as hills, cliffs, rivers, lakes. Secondly, the center of such a settlement of this type is usually occupied by the vital public building groups or squares. Because these public building groups or squares had often acted as the core of development in the early days.

Finally, the scattered settlements with strong bonds are composed of scattered traditional blocks or dwellings, and they have no boundaries. In this type of settlement, people come together for special reasons, such as a clan of blood relationship, common safety, and defense against the common enemies.

In Hunan Province, the spatial distributions of LGMTS exemplify how the ancient Chinese people create a perfect spatial layout of settlements using the advantageous conditions of environments and resources, such as regular arranging settlements with strong attractive factors and centralized-enclosed settlements, or reasonably linking the traditional rites, religions, and geomancy, such as hierarchical-institutional settlements and scattered settlements with strong bonds.

#### 4.3.2 Features of the spatial pattern atlas

In order to reveal the spatial distribution patterns and geographic rules and features of CLGTS of Hunan Province, this work established the spatial pattern atlas of LGTS (Fig. 6) according to the spatial sequence atlas and arrangement atlas. In terms of the spatial pattern atlas, the following features and rules are investigated. (i) From the types of LGTS, there are four hierarchic-institutional settlements, eleven regular arranging settlements with strong attractive factors, seven centralized-enclosed settlements, and eight scattered settlements with strong bonds, respectively. Both the sites and convenient transportation impact the site selection of traditional settlements in depth. (ii) The centralized-enclosed settlements in Hunan Province show a trend of aggregation. Such settlements mainly concentrate in the southwest and south of Hunan Province. To some extent, the aggregation distribution of the centralized-enclosed settlements in Hunan Province implies that the natural resources and landform had been fully used for building boundaries in ancient since the design and construction. Simultaneously, the boundaries deeply impact the construction and development of traditional settlements. In fact, the boundaries of settlements can enhance safety, and help people defend against the outer enemies, such as the robbers and intruders, and shelter all the residents. (iii) The regular arranging settlements with attractive factors are more helpful to the inheritance and preservation of traditional cultures than other types. In ancient times, the traditional settlements with vital communication lines could attract numerous customers and merchants and goods from different regions, such as navigable waterways, official revenues, post ways, and cross-border trade centers. At the same time, many local cultures, rites, religions, and customs from different areas are spread by different people and brought together through extensive trading activities, and this may result in the formation of new cultures and promote cultural diversities [3]. Through this case, there are many ancient temples of different religions in Jinggang Town, Furong Town, and Liye Town. (iv) Throughout Hunan Province, the scattered settlements with strong bonds merely exist in hilly or mountainous areas. On one hand, arable lands are very rare in these areas. In order to maintain more arable lands, the dwellers thus are inclined to disperse or live alone, rather than living together. On the other hand, it is difficult to build boundaries for settlements in these areas due to the surrounding complex environmental conditions, such as rugged landform or the lack of a flat plain. Hence, several scattered blocks or single traditional dwellings are usually observed in the scattered settlements with strong bonds. In this type, although the scattered blocks or dwellings are relatively independent or separate, people settle together because of strong ties such as the clan of blood relationship, the same religion or belief. (v) In Hunan Province, the spatial pattern atlas of LGTS confirms a spatial neighbouring relation between centralized-enclosed settlements and scattered settlements with strong bonds. The following two aspects can clearly address this feature. First, the centralized-enclosed settlements own a unique boundary and an obvious core of development; however, the scattered settlements with strong bonds do not. Second, LGTS of these two types can have the same cultural features such as the same settling bonds. For example, although a centralized-enclosed traditional settlement does not near to a scattered traditional settlement with strong bonds, they may have the same driving forces for development. In this case, three typical centralized-enclosed settlements showcase this feature, namely, Dayuan Village, Loutian Village, and Tantian Village. They possess the features of the clan of blood relationship and scattered blocks, and their main blocks and core of development are enclosed by their own boundaries. These three settlements have their own clan temples, and the other housings are distributed around their own clan temples respectively. Although their clan temples and boundaries deeply impact on their owned spatial image, we still cannot define them as scattered settlements with strong bonds.

Through the above, the main cultural features of the class, distribution patterns, and geographical spatial connections of traditional settlements in Hunan Province are clearly outlined according to LGMTS.

## 5 Discussion

Traditional settlements have been drawing worldwide attention due to their stark values in promoting the social and economic development. At the same time, there are still many common difficulties in preservation or development, even if the pressing need for a rational use of traditional settlements has been rapidly growing. In order to solve the current difficulties, many theoretical models or methods have been proposed during the past decades. However, there is still much room to establish a conceptual framework for addressing the key cultural features of traditional settlements from the perspective of geography. Through connecting the theory of CLGTS, this work first presented LGMTS in a bid to develop a useful method to clarify the rules or laws between the cultural factors of a certain traditional settlement and its entire spatial image.

For a certain traditional settlement, we should launch the research on a micro scale when analyzing a single cultural factor in it; on the contrary, we should consider all its cultural factors as a whole if we try to observe its whole image. To some extent, this looks like a paradox to examine the relations between a single cultural factor and the entire image of a certain traditional settlement. However, at the same time, this also significantly inspires us when we eye on the culturomics [42], because it reveals the evolutionary trend of civilization only using the frequency of words. Hence, in this work, we laid a theoretical foundation for using LGMTS (or genome maps) to determine the relations between a single cultural factor of a certain traditional settlement and its whole image. In addition, through the case study, it is clear that LGMTS is a potential way to construct a theoretical framework to reveal the natural features of traditional settlements.

As a whole, LGMTS is not so much an analysis method for the cultural factors of traditional settlements as a methodology. In this study, we developed its definition and clarified its classifications and functions. Importantly, we also showed a possible construction workflow for LGMTS through a case. Through this case, from a single one to a group of traditional settlements in a region, it reveals more important features compared with the previous research on CLGTS [15,36,37,45,50]. This hints that LGMTS makes significant progress in the theory of CLGTS.

Note that, through the instance of LGMTS in this work, three steps or stages are very important to utilize LGMTS to draw the common features and regional laws of traditional settlements for a certain region. In the first place, all the CLGTS of each traditional settlement should be accurately recognized in terms of the identification rules and methods mentioned in 2.3 and 2.4. Then, LGTS of each traditional settlement should be defined carefully according to the related CLGTS, because each traditional settlement only has a unique LGTS and this is determined by the uniqueness of CLGTS. In essence, we can use a unique code to mark and distinguish a LGTS of each traditional settlement, such as referencing the encoding method for labeling a CLGTS symbol [1]. Eventually, LGMTS is generated through organizing various LGTS and visualizing different features or rules according to the spatial differences, associations or relations of each LGTS. Of course, in the future, more generating methods and organizing approaches for LGMTS remain to be further explored when concerning different regions or countries.

## 6 Conclusions

Currently, China urgently needs to preserve and further use its tremendous traditional settlements in order to better support high-quality development, because the increasing level of urbanization and growing volume of social economy leads to a deep social reform. Therefore, it is urgent to develop new methods and theoretical models to reveal the core cultural features of traditional Chinese settlements and translate them into the information resources. Accordingly, this work presented the conception and related framework of LGMTS based on the theory of CLGTS. From this work, the following aspects are explored in depth.

To begin with, this work first presented the concept of LGMTS. This work addressed the essence of CLGTS at first. Then, this work further developed the definition of LGTS on the basis of CLGTS. Through the analogy between biological genomes and LGTS, the analysis methods and principles of biological genome maps can help to explore the cultural features and properties of traditional settlements. This work therefore developed a theoretical framework of LGMTS, determined its classifications, and further explored its promising functions.

In the second place, this work verified the application possibilities and presumable approaches of LGMTS through a case study. This work detailed the materials and constructed the corresponding LGMTS for a given region. We considered Hunan Province as the research area due to its historic and cultural depositions and also its famous “Hu-Xiang” regional cultures. We merely thought about thirty samples even though Hunan Province has hundreds of traditional settlements, because we mainly focused on the representative and integrity of historical-cultural styles and features of “Hu-Xiang” cultures. According to the related LGMTS, we recognized four types of LGTS and thoroughly examined their features. In terms of the results, LGMTS can draw the cultural rules and laws of the spatial distribution patterns of traditional settlements from the geographical perspective.

## 7 Outlook

This work developed a theoretical tool to help people grasp the nature of cultural features of traditional settlements through bridging the theoretical methods and principles of biology. Although we confirmed the potential application fields and ways of LGMTS, there are still a few issues in need of discussion.

Firstly, as an analysis method to capture the core cultural features of traditional settlements, we should explore how to introduce quantitative methods and construct related metrics. Accordingly, there are some critical attributes and features of LGMTS, such as the application conditions and expression approaches, which deserve wide attention and exploration.

Secondly, as a conceptual framework, more construction methods and workflows should be further developed if we employ LGMTS with a lot of application issues. For example, we should explore an index system for LGMTS based on GIS to measure the cultural and historic information of CLGTS when analyzing the cultural features of traditional settlements from the geographical information perspective.

Thirdly, LGMTS can reveal the key historic and cultural information of traditional settlements on a regional scale, such as spatial design models, development schematics, and survival wisdom. We therefore should develop some algorithms to mine the key historic and cultural information from LGMTS.

Eventually, LGMTS should be nourished by the quantitative analysis methods and principles from bioinformatics, and it provides a geographic information perspective to feature the knowledge of traditional settlements. This can boost the theory of CLGTS.

## CRediT authorship contribution statement

**Hu Zui:** Writing – review & editing, Writing – original draft, Resources, Methodology, Funding acquisition, Conceptualization. **Tan Min:** Writing – review & editing, Validation, Methodology.

## Data availability

Data will be made available on request.

## Funding statement

This work was supported by the 10.13039/501100001809National Science Foundation of China under grant 41771188, andthe Key Foundation of Sciences of Education Bureau of Hunan Province Government under grant21A0435, and the Foundation of Social Science Alliance of Hunan Province under grant XSP22YBC618.

## Declaration of competing interest

The authors declared no potential interest with respect to the research, authorship, or publication of this article.

And all the supporters or sponsors were acknowledged at the end of manuscript.

This work was supported by the 10.13039/501100001809National Science Foundation of China under grant 41771188, and the Key Foundation of Sciences of Education Bureau of Hunan Province Government under grant 21A0435, and the Foundation of Social Science 10.13039/100027925Alliance of Hunan Province under grant XSP22YBC618.
